# Regulation of TRAIL-Receptor Expression by the Ubiquitin-Proteasome System

**DOI:** 10.3390/ijms151018557

**Published:** 2014-10-14

**Authors:** Dhifaf Sarhan, Padraig D’Arcy, Andreas Lundqvist

**Affiliations:** Karolinska Institutet, Department of Oncology-Pathology, Stockholm S-17176, Sweden; E-Mails: padraig.darcy@ki.se (P.D.); andreas.lundqvist@ki.se (A.L.)

**Keywords:** TNF-related apoptosis-inducing ligand (TRAIL), apoptosis, cancer, ubiquitin-proteasome system (UPS), natural killer (NK) cells, T cells

## Abstract

The tumor necrosis factor (TNF)-related apoptosis-inducing ligand- receptor (TRAIL-R) family has emerged as a key mediator of cell fate and survival. Ligation of TRAIL ligand to TRAIL-R1 or TRAIL-R2 initiates the extrinsic apoptotic pathway characterized by the recruitment of death domains, assembly of the death-inducing signaling complex (DISC), caspase activation and ultimately apoptosis. Conversely the decoy receptors TRAIL-R3 and TRAIL-R4, which lack the pro-apoptotic death domain, function to dampen the apoptotic response by competing for TRAIL ligand. The tissue restricted expression of the decoy receptors on normal but not cancer cells provides a therapeutic rational for the development of selective TRAIL-mediated anti-tumor therapies. Recent clinical trials using agonistic antibodies against the apoptosis-inducing TRAIL receptors or recombinant TRAIL have been promising; however the number of patients in complete remission remains stubbornly low. The mechanisms of TRAIL resistance are relatively unexplored but may in part be due to TRAIL-R down-regulation or shedding of TRAIL-R by tumor cells. Therefore a better understanding of the mechanisms underlying TRAIL resistance is required. The ubiquitin-proteasome system (UPS) has been shown to regulate TRAIL-R members suggesting that pharmacological inhibition of the UPS may be a novel strategy to augment TRAIL-based therapies and increase efficacies. We recently identified b-AP15 as an inhibitor of proteasome deubiquitinase (DUB) activity. Interestingly, exposure of tumor cell lines to b-AP15 resulted in increased TRAIL-R2 expression and enhanced sensitivity to TRAIL-mediated apoptosis and cell death *in vitro* and *in vivo*. In conclusion, targeting the UPS may represent a novel strategy to increase the cell surface expression of pro-apoptotic TRAIL-R on cancer cells and should be considered in clinical trials targeting TRAIL-receptors in cancer patients.

## 1. Introduction

Despite recent improvements in diagnostics, clinical intervention and public awareness, cancer remains one of the leading causes of death in humans [1]. Although cancer represents a diverse spectrum of disparate diseases, all cancer cells share a common trait, namely diverse strategies to escape the body’s normal defense mechanisms. Of these, programmed cell death or apoptosis has emerged as a key process both in normal development and homeostasis and in counteracting the development of potential malignant cells. The current standard treatment of solid tumors includes surgery to remove the bulk of the tumor and subsequent radiotherapy and/or chemotherapy to kill residual cancerous cells. However a major drawback of the mainstays of standard chemotherapies is their unspecific mode of action, often causing substantial death of healthy cells [2]. Thus there is a growing recognition of the importance of targeted cancer therapies that specifically target cancerous cells while leaving normal healthy cells untouched. The tumor necrosis factor (TNF) family represents a diverse family of cell surface receptors and their corresponding ligands including; Fas ligand (FasL)/First apoptosis signal Fas, TNF-related apoptosis-inducing ligand (TRAIL)/TRAIL-Rs, Cluster of differentiation CD40/CD40L, 4-1BB (CD137)/4-1BBL (CD137 ligand), TNF-α/TNF-receptor1 (TNF-RI), and Lymphotoxin-alpha (LTα)/TNF-RII/LTα-receptor β (LTαRβ) that directly or indirectly regulate apoptosis [3]. Unlike many chemotherapeutic drugs, the TNF-family members, in particular TRAIL, have the ability to induce apoptosis in transformed but not normal cells [4,5,6]. As such, several clinical studies have explored the efficacy of targeting TRAIL-receptors on tumor cells, either as single treatments or in combination with other chemotherapeutic drugs [7]. Activated T and natural killer (NK) cells express high levels of TRAIL ligand that can potently kill tumor cells expressing TNF-related apoptosis-inducing ligand-receptor (TRAIL-R) [8,9], suggesting that immune-modulating therapies may be of particular benefit for treating cancer. However, tumor cells can also down-regulate the expression of TRAIL-receptors and acquire resistance to TRAIL-mediated apoptosis. As such, combination therapies that can increase TRAIL-R expression or prevent down-regulation would be of therapeutic benefit. Here we review the molecular mechanisms of TRAIL-receptor expression in transformed cells and how the immune system can be harnessed to eliminate TRAIL-receptor positive tumor cells.

## 2. Modulation of Tumor Necrosis Factor (TNF)-Related Apoptosis-Inducing Ligand (TRAIL)-Receptors in Cancer

### 2.1. TRAIL-Receptor Expression in Healthy vs. Malignant Tissue

In the 1990s the TRAIL-R was initially identified and characterized [10]. The TRAIL-R family is composed of two pro-apoptotic death receptors (DRs), TRAIL-R1 and TRAIL–R2, and two anti-apoptotic membrane receptors, TRAIL-R3 and TRAIL-R4. Unlike the pro-apoptotic receptors, TRAIL-R3 and TRAIL-R4 do not contain the intercellular death domain (DD) required for apoptosis induction and as such act as decoy receptors to dampen TRAIL-induced death signaling [11]. The TRAIL induced apoptotic pathway has been suggested to be a key component in discriminating between transformed and non-transformed cells. The presence of decoy receptors adds an additional layer of complexity to TRAIL-mediated apoptosis by competing with the pro-apoptotic TRAIL receptors for TRAIL ligand and/or by activating cell survival pathways via NF-κB (nuclear factor κ-light-chain-enhancer of activated B cells), ERK (extracellular signal-regulated kinases), and p38 [12,13]. In contrast to normal cells, tumor cells preferentially express higher levels of the death-inducing TRAIL-receptors making them more susceptible to TRAIL-mediated apoptosis [14]. The fact that TRAIL preferentially induces apoptosis of transformed cells and that TRAIL-deficient mice show increased levels of tumorigenesis suggests targeting TRAIL-mediated signaling as a potential anti-cancer strategy [4,15]. Deregulation of TRAIL-R expression is frequently observed in tumors where the increased expression of TRAIL-R1 and TRAIL-R2 is correlated with early tumor stage [16]. Conversely down regulation of TRAIL-R has been observed in colorectal cancer patients with more advanced tumor grades [17]. Similarly, loss of membrane-bound TRAIL-R1 and TRAIL-R2 has been correlated, with poor prognosis in pancreatic cancer patients [18]. Such data suggests that the expression of TRAIL-Rs may serve as a useful prognostic biomarker in staging disease progression. However the picture is by no means definitive, since higher TRAIL-R1 expression has also been correlated with a more aggressive phenotype in patients with invasive ductal carcinoma [19].

### 2.2. TRAIL-Ligand vs. Fas Ligand (FasL)

Targeted activation of death receptors has long been proposed as an anti-cancer therapy. Initially Fas was examined as a potential target; however data from animal studies has generated safety concerns in targeting Fas and other death receptors [20,21]. However due to the reasons discussed below, targeting TRAIL-R may not be associated with the dose-limiting toxicities observed in the pre-clinical studies with Fas. Similar to TRAIL, FasL is a member of the TNF-family that induces apoptosis upon binding to its receptor (CD95). While FasL expression is tightly regulated and only transiently expressed on activated cells, TRAIL mRNA is constitutively expressed in a wide range of tissues [22]. TRAIL expression is found in fetal lungs, liver, thymus, placenta, ovary, small and large intestine, prostate, thyroid, and heart [14,22]. TRAIL expression has been detected in activated and resting B lymphocytes, activated T cells, NK cells, monocytes, macrophages, and dendritic cells [23]. In spite of this broad spectrum of expression, TRAIL-mediated apoptosis is highly regulated and selectively induced in tumor but not normal cells [4,5]. While, the physiological function of TRAIL in normal cells is not fully known, FasL is a well-established regulator of immune homeostasis [24,25]. Deregulated FasL expression is associated with hyper immune responses and toxicity. Compared with TRAIL, FasL expression is under much stricter control including; regulated intracellular storage and shedding by metalloproteases [26,27,28]. TRAIL is expressed by a variety of human tissues and multiple cell types within the immune system. Levels of TRAIL expression is determined by the proinflammatory cytokine profile. Cytokines such as IFN-α, IFN-β, and IFN-γ stimulate TRAIL expression. In addition, IL-2, IL-7, and IL-15 trigger the expression of TRAIL in NK cells and LPS potentiates the cytotoxicity of macrophages and monocytes through increased TRAIL expression [26,29,30,31]. The role of IFN-γ in induction of TRAIL expression in NK cells has also been demonstrated. Similarly, only IFN-γ-stimulated monocytes, dendritic cells, and CD4 T lymphocytes express TRAIL [32]. Both FasL and TRAIL play a central role in the induction of the cytotoxicity of T and NK cells in infectious diseases and cancer [33,34]. However, a major drawback of targeting Fas as an anti-cancer therapy is that in contrast to TRAIL-R, Fas displays broad tissue expression in both normal and tumor cells raising the risk of severe toxicity from Fas-based therapies. For example, Fas is generally expressed in hepatocytes, cholangiocytes, sinusoidal endothelial cells, stellate cells, and Kupffer cells [35], which makes these sites more susceptible to FasL. Several studies have reported a correlation between tissue damage and elevated FasL expression levels [36]. The sFasL (soluble FasL) levels of sera in patients with Stevens-Johnson syndrome or toxic epidermal necrolysis are significantly increased before development of skin detachment, mucosal lesions, or both [37]. Moreover, a rapid reversal of the disease progression was observed in toxic epidermal necrolysis patients treated with antibodies blocking the interaction between Fas-FasL [38]. In addition, a massive hepatic apoptosis has been observed in an acute liver failure mouse-model, after Fas ligand treatment [39]. On the other hand, few reports have shown a toxicity profile in healthy tissue that correlates with TRAIL engagement. Given the importance of targeting the correct cytotoxic molecule in cancer, Fas might not be an optimal target.

### 2.3. TRAIL-Induced Apoptosis Pathways

**Figure 1:**
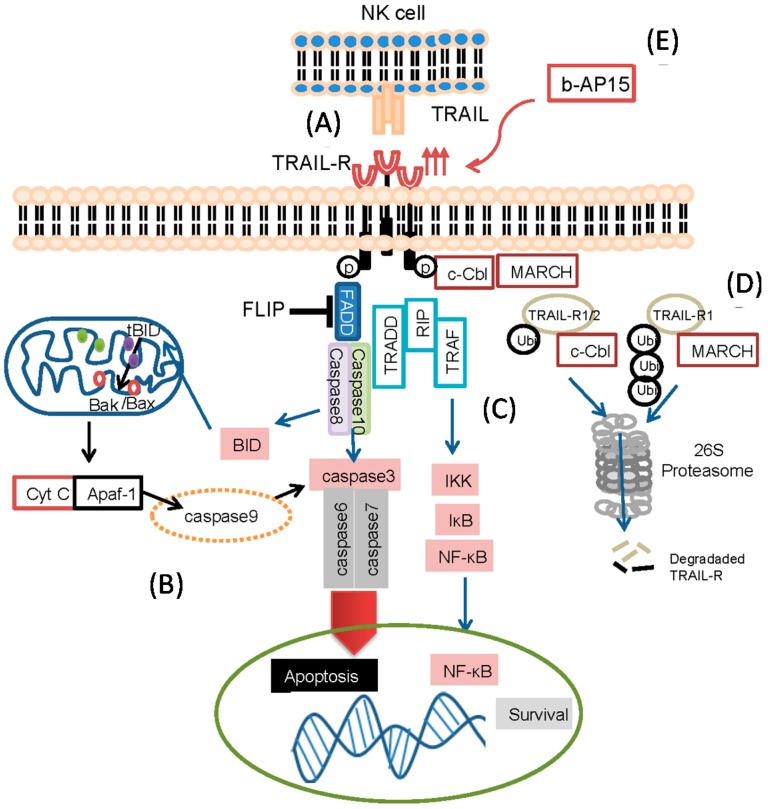
Tumor necrosis factor (TNF)-related apoptosis-inducing ligand (TRAIL)-pathway. The fate of ligating apoptosis-inducing TRAIL-receptors. (**A**) TRAIL-receptors on the surface of cells can be ligated by TRAIL expressed on activated lymphocytes such as natural killer (NK) cells; (**B**) TRAIL mediates apoptosis by activating the caspase-cascade or the mitochondrial pathway by the release of cytochrome c; (**C**) TRAIL- can also mediate survival of the cells by activating the NF-κB pathway; (**D**) The ubiquitin-proteasome system can assist in the degradation of TRAIL-Rs; and (**E**) Exposure of tumor cells to the DUB inhibitor b-AP15 (3,5-bis[(4-nitrophenyl)methylidene]-1-prop-2-enoylpiperidin-4-one), results in the up-regulation of TRAIL-R2. Acronyms: FLIP (FLICE-like inhibitory protein), FADD (Fas-Associated protein with Death Domain), TRADD (Tumor necrosis factor receptor type 1-associated death domain protein), TRAF (TNF receptor-associated factor), RIP (Receptor-Interacting Protein), Cyt C (cytochrome C), Apaf-1 (Apoptotic Protease Activating Factor 1), c-Cbl (Casitas B-lineage Lymphoma), MARCH (membrane associated RING-CH), tBID (truncated BID), ubi (ubiquitination).

Programmed cell death is regulated by several different signaling pathways. A distinct method used by NK- and T- cells to target tumor cells is TRAIL-mediated apoptosis [40,41,42,43,44]. Upon ligation of death receptors by their corresponding ligands or agonistic antibodies, the extrinsic apoptotic pathway is initiated through oligomerization of the death receptors [45]. When TRAIL binds to the pro-apoptotic receptors TRAIL-R1, TRAIL-R2, TRAIL promotes recruitment of Fas-associated protein with death domain (FADD) and assembles the death-inducing signaling complex (DISC), leading to pro-caspase-8 activation and initiation of apoptosis [46,47,48] (Figure 1A). To avoid inadvertent intracellular damage, the apoptotic cascade is highly regulated. In particular, isoforms of the cellular FLICE-like inhibitory protein (c-FLIP) inhibit the pro-apoptotic process. c-FLIP competes with caspase-8 for binding to FADD, preventing the downstream activation of the apoptotic cascade [49,50,51]. The extrinsic apoptosis pathway is orchestrated by the activation of caspase-8 and caspase-10. Catalytic cleavage of caspase-8/10 propagates the signal further to directly activate and cleave the downstream members caspase-3, caspase-6 and caspase-7, the so-called effector-caspases, or by cleaving BID to its truncated form (tBID) following translocation to mitochondria [52]. Through a tightly regulated process by pro- and anti-apoptotic members of the B cell lymphoma 2 (BCL-2) family, mitochondrial outer membrane permeabilization (MOMP) is induced and cytosolic cytochrome c is released [53]. Subsequently, cytosolic cytochrome c binds to apoptotic peptidase activating factor 1 (APAF-1) and activates caspase 9, which in turn, cleaves and activates effector-caspases [52], eventually leading to the phagocytic recognition and clearance of the dying cell (Figure 1B).

In addition to triggering caspases, signaling of TRAIL-R1 and -R2 can also result in the recruitment of receptor-interacting protein (RIP) to TRADD. RIP can then activate NF-κB-inducing kinase (NIK), which phosphorylates IKK (inhibitor of nuclear factor κ-B kinase subunit β) leading to proteasome degradation of phosphorylated IκB as well as nuclear translocation and anti-apoptosis signaling of NF-κB. It was recently shown that inhibition of RIP enhances TRAIL-induced apoptosis in pancreatic cancer cells [54] (Figure 1C).

### 2.4. TRAIL Targeted Therapies

Given its safety profile and favorable tumor specificity compared to other TNF family members, TRAIL targeting is being increasingly explored. From a high-throughput chemical screen for compounds that promote cell death in synergy with TRAIL, Wang *et al.* identified bioymifi ((*Z*)-5-(5-[(3-[4-bromophenyl]-2-imino-4-oxothiazolidin-5-ylidene)methyl]furan-2-yl)isoindoline-1,3-dione) as a Smac (Second mitochondria-derived activator of caspase) mimetic which potentiates TRAIL-induced apoptosis. Bioymifi promoted the recruitment of DISC, FADD, TRADD and RIPK1 followed by caspase-8 dependent apoptosis and cell death. Notably, knock down of TRAIL-R2 in human glioblastoma cells abolished the apoptotic effect of bioymifi, supporting the central role of TRAIL-mediated killing of tumor cells [55]. Other studies have focused on augmenting the susceptibility to TRAIL-targeting via up-regulation of the cell surface expression of TRAIL-receptors on tumor cells. Wang *et al.* showed that stabilizing p53 in mutated tumor cell lines by small molecules, including, CP-31398, PRIMA1, and Nutlin, was accompanied by increased expression of TRAIL-R2, as well as a reduced tumor-burden *in vivo* models [56]. In addition, we, and others, have found that exposure to doxorubicin resulted in the up-regulation of TRAIL-R2, down-regulation of c-FLIP and increased sensitivity to TRAIL-induced apoptosis in several different tumor cell lines *in vitro* [57,58]. Also, Borbone *et al.*, have shown that usage of histone deacetylase inhibitors induces proteasome-dependent inhibition of TRAIL degradation which results in increased specific apoptosis in thyroid cancer cells [59].

A major mechanism used in tumor cell targeting by NK cells and T-cells utilizes TRAIL-mediated apoptosis [40,41,42,43,57]. Natural killer (NK) cells have a spontaneous cytotoxic capacity against tumors and several studies have investigated the prognostic significance of infiltrating NK cells in solid tumors. In a study by Coca *et al*., intratumoral NK cell infiltration was investigated in 186 patients with adenocarcinoma, with NK infiltration positively correlated with patient survival [60]. Furthermore the molecular signatures of activated NK cells in breast cancer patients could predict relapse-free survival [61]. Given the importance of NK cells and TRAIL signaling in cancer, several research groups have investigated the potential of NK cells expressing high levels of TRAIL to target tumors. A major drawback is however that effector cells such as NK cells tend to lack, or express very low levels of TRAIL ligand. Indeed, we recently demonstrated that peripheral blood NK cells express low levels of TRAIL and are unable to kill TRAIL-sensitive tumors [62]. However activation with IL-2 or IL-15 resulted in increased TRAIL expression by NK cells, although the levels were highly variable [63]. We found that exposure of NK cells to bisphosphonate zoledronic acid resulted in increased expression of TRAIL by NK cells, and augmented TRAIL-mediated cytotoxicity against tumors *in vitro* and *in vivo* [64].

Other investigators have overcome the lack of TRAIL expression on effector cells by utilizing recombinant proteins as a strategy to target tumors via the TRAIL pathway. Treatment with recombinant TRAIL (dulanermin) has been explored in clinical trials [65,66,67]. A significant increase in serum caspase 3/7 levels were detected in cohorts of colorectal and sarcoma patients after receiving dulanermin. Also in the last decade, TRAIL-specific agonistic antibodies targeting TRAIL-receptors, mapatumumab (anti-TRAIL-R1) and lexatumumab (anti-TRAIL-R2) have been evaluated in clinical trials. In a cohort of forty patients with relapsed or refractory non-Hodgkin's lymphoma, 7.5% of the patients experienced clinical responses with complete or partial responses reported following treatment with mapatumumab. These drugs are generally well tolerated, with no patients experiencing drug-related hepatic or other dose-limiting toxicity [66,67].

### 2.5. Resistance to TRAIL-Targeted Therapies

Although the results of the above clinical trials suggest the potential of targeting TRAIL as an anti-cancer therapy, several mechanisms of resistance to TRAIL-mediated apoptosis have been reported. Tumor cells can up-regulate the expression of decoy receptors to down-regulate apoptotic signaling by competing for TRAIL on effector lymphocytes [55,68]. In addition, loss of TRAIL-R1 and -R2 expression on tumor cells can lead to TRAIL resistance [69]. One potential resistance mechanism observed in tumor cells involves defects associated with the post-translational regulation of TRAIL-R1/2. Expression of the enzyme *N*-ethylgalactozamine transferase 14, that mediates clustering of the receptors upon ligation, has been correlated with TRAIL sensitivity in number of tumor cell lines, Loss of this enzyme is often associated with a decreased response to TRAIL stimulation [70]. Several proteins that mediate apoptosis downstream of the TRAIL pathway also display altered expression levels. For example increased methylation of the caspase-8 promoter has been associated with reduced caspase-8 expression levels [71,72]. Alternatively increased expression of the anti-apoptotic protein c-FLIP can function upstream of caspase-8 to decrease apoptosis signaling [73]. Interestingly c-FLIP shows variable expression depending on tumor type and may represent a key mechanism of TRAIL-specific tumor escape [74,75,76]. Alternative mechanisms for resistance to TRAIL-mediated apoptosis includes the overexpression of the anti-apoptotic proteins Bcl-2 and Bcl-XL, both of which function to prevent mitochondrial release of cytochrome C and activation of the intrinsic apoptosis pathway [77,78].

### 2.6. The Role of Ubiquitin in Regulating TRAIL Receptors

Since its initial description over 30 years ago, the ubiquitin-proteasome system (UPS) has emerged as a key regulator of numerous cellular processes. The UPS is composed of a multi-enzymatic cascade that results in the covalent attachment of the small molecule ubiquitin to lysine residues within target proteins. A large and diverse family of ubiquitin E3 ligases confers substrate specificity and catalyzes the formation of isopeptide bonds between ubiquitin and the target proteins. An additional ubiquitin code exists whereby the nature of the ubiquitin linkages has a direct consequence on outcome. Traditionally ubiquitin chains composed of ubiquitin linked via internal K48 linkages target conjugated proteins for degradation by the proteasome (a large multi-protein complex that hydrolyzes ubiquitin tagged proteins into small peptide fragments), whereas K63 linked chains are involved in signaling and trafficking events [79]. Multiple cell surface receptors are subject to regulation by the UPS and it would appear that the TRAIL-R family is no exception (Table 1).

The E3 ligase c-Cbl has been shown to directly regulate TRAIL-R ubiquitination following receptor activation [80]. Stimulation with TRAIL ligand induced c-Cbl-mediated mono-ubiquitination of TRAIL-R1 and TRAIL-R2 resulting in the degradation of internalized receptors by the proteasome. The authors propose a model whereby c-Cbl contributes to early phase TRAIL resistance by reducing TRAIL-R levels following stimulation. Recently a second group demonstrated differential regulation of TRAIL-R expression by members of the membrane associated RING-CH (MARCH) family of ubiquitin ligases [81]. Exogenous overexpression of MARCH-1, MARCH-2 or MARCH-9, leads to the preferential poly-ubiquitination of TRAIL-R1 over TRAIL-R2 on a conserved lysine residue in the juxta-membrane domain of the receptor. Surprisingly MARCH-mediated ubiquitination of TRAIL-R1 led to receptor internalization and degradation by the lysosomal, as opposed to the proteasomal pathway (Figure 1D). Importantly, MARCH overexpression was correlated with increased resistance to apoptosis following TRAIL stimulation. To date, only c-Cbl and the MARCH E3 ligases have been identified as direct mediators of TRAIL-R cell surface expression, however, based on the presence of putative ubiquitin sites in the protein sequence, it is highly probable that additional E3 ligases will be identified in the future.

In addition to direct effects on TRAIL-R expression and levels, several independent reports have also demonstrated a role for ubiquitin in the signaling cascade downstream of TRAIL activation. In particular caspase-8 activation appears to be regulated by multiple components of the UPS. Jin *et al.*, identified caspase-8 as a component of the DISC complex whose activity is controlled by both ubiquitination and deubiquitination events [82]. Ubiqutination of procaspase-8 by the E3 ligase cullin-3 (CUL3) creates binding sites for the scaffold protein p62/SQSTM. This association of p62 promotes the formation of micro-clusters of procaspase-8 that facilitates the processing of the pro-caspase into the catalytically active form. Conversely the authors identified the A20 as a deubiquitinase (DUB) that down-regulates caspase-8 activity by removing the ubiquitin chains required for procaspase-8 clustering. Interestingly A20 has a duplicitous role in the regulation of caspase-8 activity and can function as both an E3 ligase and a DUB. Overexpression of A20 in glioblastoma cells led to K63-linked ubiquitination of RIP1, which interacts with protease domains of procaspase-8 and interferes with activation and downstream signaling [83]. Whereas CUL3-mediated ubiquitination promotes caspase-8 activation, several other E3 ligases have been identified as caspase-8 inhibitors. The E3 ligase HECTD3, inhibits TRAIL-induced apoptosis by directly modifying caspase-8 with K63 linked poly-ubiquitin [84]. This observation is of clinical interest since HECTD3 is frequently overexpressed in a subset of breast cancers, thus may confer resistance to TRAIL-based therapies. Other recent additions to the repertoire of mediators in caspase-8 activation are the E3 ligases WWP1, Siah2 and POSH that down-regulate caspase-8 activation and TRAIL-induced apoptosis, however whether this is due to direct ubiquitination of caspase-8 remains to be determined [85,86]. In addition, c-FLIP steady state levels are regulated by the E3 ligase Itch that targets FLIP for proteasomal degradation [87].

Based on the diverse roles of the UPS in regulating TRAIL signaling, it is of no surprise that proteasome inhibitors have been suggested as potential treatments to increase TRAIL-mediated activities in the clinical setting [88]. Several reports have shown that treatment with proteasome inhibitors can up-regulate the cell surface expression of TRAIL-R receptors leading to increased sensitization to TRAIL-induced apoptosis [89,90,91,92]. The clinically used proteasome inhibitor, bortezomib has been shown to increase sensitivity to TRAIL via up-regulation of TRAIL-R2 expression levels in NSLCC cells [89]. Importantly this effect was refractory to the expression of FLIP and survivin, suggesting co-treatment may be an effective strategy to overcome TRAIL resistance. In prostate cancer, proteasome inhibitors also induced the up-regulation of TRAIL-R2 and sensitivity to TRAIL-induced apoptosis, suggesting that the combination of proteasome inhibitors and TRAIL is a novel therapeutic approach in treating TRAIL resistant tumors [92]. The mechanisms by which proteasome inhibitors up-regulate TRAIL-R expression appears to be complex. A block in ubiquitin mediated proteolysis by proteasome inhibitors would be expected to increase TRAIL-R levels. However several reports have also shown that proteasome inhibitors can alter TRAIL-R expression by alternative mechanisms. For example proteasome inhibition induces p53 accumulation that binds to response elements within the intronic sequence of the *TRAIL-R2* gene leading to increased gene expression [91]. Proteasome inhibition also induced the association of the RNA stabilizing protein HuR with the 3'UTR of TRAIL-R2 mRNA leading to an increased half-life [90].

Considering the importance of the TRAIL pathway in inhibiting cancer cell survival, the rational design of small molecule inhibitors targeting specific E3 ligases or DUBs that regulate components of the pathway may be a potential therapeutic strategy.

**Table 1:** Ubiquitin ligases and regulation of the TRAIL signaling pathway.

Ligase	Target	Outcome
c-Cbl	TRAIL-R1 and R2	Mono-ubiquitination and degradation of internalized receptors by the proteasome
MARCH-1, -2, and -9	TRAIL-R1 and R2	Poly-ubiquitination, internalization and degradation of internalized receptors by the lysosomal pathway
A20	Procaspase-8	K63-linked ubiquitination of RIP1 and down-regulation of caspase-8 activity
HECTD3	Caspase-8	Facilitates survival by promoting K63-linked polyubiquitination of caspase-8
Itch	FLIP	Target FLIP for proteasomal degradation
CUL3	Procaspase-8	Ubiqutination of procaspase-8 to create binding sites for the scaffold protein p62/SQSTM

Cbl (Casitas B-lineage Lymphoma), MARCH (members of the membrane associated RING-CH), HECTD3 (Homologous to the E6-associated protein carboxyl terminus domain containing 3, Itch (E3 ubiquitin-protein ligase Itchy homolog), CUL3 (Cullin-based E3 ligases), FLIP (FLICE-like inhibitory protein).

### 2.7. Enhancing TRAIL-Mediated Apoptosis by Targeting Ubiquitin-Proteasome System (UPS)

Given the role of the UPS on regulating TRAIL-R expression and stability, several studies have evaluated the role of proteasome inhibitors on TRAIL-R expression. The 20S core proteasome inhibitor bortezomib affects many anti- and pro-apoptotic proteins, and induces cytotoxicity through c-Jun NH_2_-terminal kinase/caspase activation in various types of tumors [93,94]. Bortezomib and TRAIL act in concert to cause accumulation of tBID, the active cleavage product of BID and induce mitochondrial dependent apoptosis of tumor cells [95].

Importantly, treatment with bortezomib increases the expression of TRAIL-R2 on tumor cells resulting in increased susceptibility to killing by NK cells [96]. In an ongoing clinical trial, we found that using highly activated NK cells following bortezomib treatment was well tolerated [97], suggesting that bortezomib is a good combinational treatment for immunotherapy.

We recently identified b-AP15 (3,5-bis[(4-nitrophenyl)methylidene]-1-prop-2-enoylpiperidin-4-one) as a novel inhibitor of the UPS that blocks the deubiquitinating activity of the proteasome. b-AP15 inhibits two proteasome-associated DUBs, USP14 and UCHL5, resulting in a rapid accumulation of high molecular weight ubiquitin conjugates and a functional proteasome shutdown [98,99]. Using a panel of cancer cell lines we found that short exposure to b-AP15 resulted in the up-regulation of TRAIL-R2 and an increase in TRAIL-specific targeting of tumor cells by NK cells (Figure 1E). In addition, we found b-AP15 not only increased the expression of TRAIL-R2, it also decreased the expression levels of c-FLIP, which could enhance TRAIL-targeting of tumor cells [8]. Other studies have demonstrated that degradation of c-FLIP sensitizes tumor cells to TRAIL-mediated apoptosis. The NEDD8-activating enzyme inhibitor, MLN4924, was recently shown to cooperate with TRAIL to augment apoptosis through facilitating c-FLIP degradation in head and neck cancer cells [100]. Furthermore, treatment with troglitazone sensitized glioma cells to TRAIL-induced apoptosis regulated by proteasome-dependent degradation of FLIP and ERK1/2-dependent phosphorylation of BAD [101]. Unlike bortezomib, that renders tumor cells resistant to killing by tumor-specific T cells due to impaired antigen processing and presentation, we found that b-AP15 treatment sensitized tumor cells to TRAIL-mediated killing by tumor-specific T cells [102]. Although the exact mechanism of how b-AP15 enhances T-cell mediated killing is unknown, we postulate that b-AP15 does not inhibit antigen processing by the immunoproteasome and the presentation of antigenic peptides to T cells [8].

Considering the capacity of b-AP15 to specifically enhance the expression of TRAIL-R2 on tumor cells, we suggest the combination of b-AP15 and cellular therapy with activated NK- and T-cells as a potential therapeutic strategy for cancer patients.

## 3. Concluding Remarks

Tumor cells preferentially express apoptosis-inducing TRAIL-Rs on the cell surface, providing a tempting therapeutic target for the development of immunomodulating therapies for the treatment. Importantly normal cells tend to express lower levels of TRAIL-R and/or increased expression of TRAIL decoy receptors, which render the cells refractory to TRAIL-induced apoptosis. Although clinical studies on targeting TRAIL-Rs has provided some evidence of clinical efficacy, better understanding of how TRAIL-Rs are regulated and how these receptors can be induced, is needed. Based on recent evidence implicating the UPS in regulating TRAIL-R activity, inhibitors of UPS activity may represent a novel strategy to increase TRAIL-R expression and mediate NK and T cell mediated killing of cancer cells.
